# Oral health and public speaking attitudes after dental implant treatment in septuagenarians: a comparative study

**DOI:** 10.3389/fpubh.2026.1840468

**Published:** 2026-06-11

**Authors:** Irfan Ustundag, Selman Bolukbasi, Bahadir Sancar, Recep Akmese, Agit Simsek

**Affiliations:** 1 Department of Oral and Maxillofacial Surgery, Faculty of Dentistry, Inonu University, Malatya, Türkiye; 2 Department of Gerontology, Faculty of Health Sciences, Balikesir University, Balikesir, Türkiye; 3 Department of Speech and Language Therapy, Faculty of Health Sciences, Inonu University, Malatya, Türkiye

**Keywords:** aged, attitude to health, communication, dental implants, oral health, septuagenarian

## Abstract

**Background:**

Dental implant treatment is increasingly used in older adults to restore oral function and psychosocial well-being. However, its relationship with oral health attitudes and public speaking attitudes remains unclear, particularly in adults aged ≥70 years. This study examined these associations in implant-treated adults aged ≥60 years, with a focus on septuagenarians.

**Methods:**

This cross-sectional study included 277 implant-treated adults aged ≥60 years from the Department of Oral and Maxillofacial Surgery, Inonu University Faculty of Dentistry, including 85 aged ≥70 years and 192 aged 60–69 years. Participants completed the Oral Health Attitude Scale, the Turkish Public Speaking Attitude Scale, and a sociodemographic questionnaire. Nonparametric tests, Spearman correlation, and multivariate linear regression were used for analysis.

**Results:**

Oral health attitudes showed significant positive correlations with public speaking enjoyment and perceived importance, and a negative correlation with speech anxiety. These associations were stronger in the septuagenarian subgroup. In the total sample, prior removable denture use, implant opinion, psychiatric medication use, and education level independently predicted oral health attitudes. Public speaking enjoyment was negatively associated with prior denture use and psychiatric medication use in the total sample, whereas positive implant opinion and male gender were significant correlates in septuagenarians. Regression models for speech anxiety were not significant.

**Conclusion:**

Positive oral health attitudes were associated with greater public speaking confidence in implant-treated older adults, particularly in those aged ≥70 years. Prior denture use and psychiatric medication use were negatively associated factors, whereas positive implant opinion was independently associated with higher public speaking enjoyment scores in septuagenarians.

## 1 Introduction

The rapid increase in the proportion of older adults globally, including in Türkiye, has underscored the need for age-specific approaches to health promotion (1, 2). Oral health in aging is not limited to masticatory function; it profoundly influences speech competence, social engagement, and overall psychosocial well-being (1, 3). The World Health Organization (WHO) has identified oral health maintenance in older adults as an integral component of healthy aging strategies (2).

Dental implant treatment has become the most effective and widely adopted method for rehabilitating tooth loss in older individuals (4). Beyond restoring masticatory function, implants improve aesthetics, voice quality, and the capacity for self-expression in social settings (5). Evidence indicates that successful oral rehabilitation strengthens social connectedness and mitigates the psychological burden of tooth loss, including social isolation, low self-esteem, and communication anxiety (6, 7).

An individual’s attitude toward oral health plays a decisive mediating role in translating clinical gains into social participation (8). Public speaking behavior is directly linked to self-confidence, social anxiety, and the individual’s mode of self-expression. Despite the extensive literature on the physical outcomes of implant therapy, studies examining the relationship between oral health attitudes and public speaking confidence remain scarce-particularly in older populations. This gap highlights the need for multidisciplinary approaches integrating gerodontology, audiology, and speech-language therapy (9). The Oral Health Attitude Scale (OHAS) was selected because it captures multidimensional cognitive and behavioral orientations toward oral health maintenance, including sensitivity to oral symptoms, perceived importance of oral care, and social influence on oral health behaviors—domains that are theoretically expected to mediate the translation of implant-related physical restoration into psychological and communicative outcomes. The Public Speaking Attitude Scale (PSAS) was selected because it assesses three functionally distinct dimensions of public speaking engagement—enjoyment, perceived importance, and anxiety—that collectively reflect the degree to which an individual embraces, values, and experiences distress in oral communicative situations. In geriatric implant patients, whose social functioning and self-expression are directly influenced by oral rehabilitation, this combination of instruments allows the pathway from oral health attitude to communicative confidence to be traced across multiple dimensions simultaneously. Specifically, we hypothesized that individuals with more positive oral health attitudes following implant treatment would report greater enjoyment and perceived importance of public speaking, and lower communication anxiety, with these associations being particularly pronounced among the oldest-old (aged ≥70 years), in whom oral rehabilitation-related psychosocial gains have been shown to be mediated predominantly by perceptual rather than biological factors.

To the best of our knowledge, this is the first study to comparatively examine the relationship between oral health attitudes and public speaking attitude among implant-treated older adults, with a specific focus on the septuagenarian subgroup (aged ≥70 years). The septuagenarian subgroup was identified as the primary subgroup of interest because adults aged ≥70 years represent the fastest-growing segment of implant recipients globally, yet the psychosocial dynamics of implant treatment in this specific age stratum remain underexplored relative to the broader ≥60-year population. Importantly, the total sample (*N* = 277) encompasses both age groups to allow comparative analyses, and all primary findings are reported for the full sample; the septuagenarian subgroup serves as a pre-specified focus of exploratory subgroup analysis rather than the sole unit of investigation. The three subscales of the PSAS were analyzed separately to allow multidimensional interpretation of the findings. The central hypothesis was that higher oral health attitude scores would be associated with greater public speaking confidence and lower communication anxiety following dental implant treatment. We anticipate that these findings will offer a novel perspective on the psychosocial and communicative outcomes of implant therapy, addressing a gap in the gerodontology and communication sciences literature.

## 2 Methods

### 2.1 Study design and setting

This cross-sectional study was conducted at the Department of Oral and Maxillofacial Surgery, Inonu University Faculty of Dentistry (Malatya, Türkiye). The study was designed and conducted in accordance with the principles of the Declaration of Helsinki. Ethical approval details are provided in the Ethical Considerations section.

### 2.2 Participants

The study population comprised adults aged ≥60 years who had received dental implant treatment at the abovementioned clinic within the preceding 6 years. Inclusion criteria were: (i) age ≥60 years, (ii) at least one dental implant placed at the study clinic, and (iii) voluntary written informed consent. Exclusion criteria included a history of psychiatric or neurological disorders (eg, dementia, schizophrenia) or cognitive impairment precluding questionnaire completion.

A total of 277 participants meeting all inclusion criteria were enrolled: 85 (30.7%) in the septuagenarian subgroup (≥70 years) and 192 (69.3%) in the 60-69-year group. The sample size was considered adequate for regression analyses based on the recommended observations-per-predictor ratio. *Post hoc* power analyses (Cohen’s *f*^2^, *α* = 0.05, 8 predictors) confirmed statistical power of 1 − *β* ≥ 0.980 for all significant models in the total sample, and 1 − *β* = 0.939–1.000 for significant models in the septuagenarian subgroup. For the non-significant PSAS-Anxiety models, statistical power was limited (1 − *β* = 0.283–0.512), suggesting that low effect size may have contributed to non-significance. A minimum sample of *N* = 109 is required to achieve 80% power for a medium effect (*f*^2^ = 0.15) with 8 predictors; the total sample substantially exceeded this threshold.

### 2.3 Measures

#### 2.3.1 Sociodemographic questionnaire

A structured 22-item sociodemographic questionnaire was used to collect data on age, gender, education level, number of implants, years since implant completion, prior removable denture use, psychiatric medication use, and frequency of social interaction.

#### 2.3.2 Oral health attitude scale (OHAS)

The OHAS was developed by Fidan et al. (10) to assess oral health attitudes. The scale underwent exploratory factor analysis (*n* = 470) and confirmatory factor analysis (*n* = 300) during its development. It consists of 41 items rated on a 5-point Likert scale (1 = Strongly Disagree to 5 = Strongly Agree), with no reverse-scored items. Total scores range from 41 to 205, with higher scores indicating more positive oral health attitudes. The scale comprises six subscales: Sensitivity (12 items), Importance (6 items), Avoidance of Harmful Substances (7 items), Tendency Toward Products and Activities (6 items), Awareness (6 items), and Social Influence (4 items). Internal consistency was excellent (Cronbach *α* = 0.92 for the total scale; subscale *α* range: 0.77–0.89). The Spearman-Brown split-half reliability coefficient was 0.91. Confirmatory factor analysis demonstrated acceptable model fit (RMSEA = 0.080; GFI = 0.90; CFI = 0.91; NNFI = 0.90).

#### 2.3.3 Public speaking attitude scale

The PSAS was originally developed by Brown and Welch (11) to assess public speaking attitudes in higher education students, with psychometric properties established through exploratory and confirmatory factor analyses. The Turkish adaptation was performed by Aydoğan and Çelik (12), with written permission obtained from the original authors. The scale consists of 16 items rated on a 5-point Likert scale (1 = Strongly Disagree to 5 = Strongly Agree) and comprises three subscales: Enjoyment (items 1–8; score range 8–40), Perceived Importance (items 9–13; score range 5–25), and Anxiety (items 14–16; score range 3–15). Anxiety items are reverse-scored; thus, higher scores indicate lower anxiety. In the Turkish adaptation, Cronbach *α* coefficients were 0.95 (Enjoyment), 0.85 (Perceived Importance), and 0.91 (Anxiety). Confirmatory factor analysis demonstrated excellent model fit (*χ*^2^/df = 1.80; RMSEA = 0.039; CFI = 0.99; TLI = 0.99; SRMR = 0.039); factor loadings ranged from 0.71 to 0.96. Given the nonsignificant correlation between the Perceived Importance and Anxiety subscales (*r* = −0.058), a total score is psychometrically inappropriate; therefore, subscales were analyzed separately (12).

### 2.4 Data collection

Data were collected through face-to-face interviews following a standardized administration sequence and avoiding leading prompts. Responses were entered into a Google Forms-based digital data collection system by the researchers. Written informed consent was obtained from all participants prior to data collection; illiterate participants provided witnessed thumbprint consent (see Ethical Considerations). To minimize interviewer bias, all researchers who conducted the interviews underwent a standardized calibration session prior to data collection; this session included review of item wording, practice administration, and discussion of response prompting protocols. Interviews were conducted in a private, quiet consultation room within the clinic to ensure confidentiality and reduce environmental distractions. The mean duration of each interview session was approximately 25–30 min. To reduce the risk of response fatigue, particularly given the 79-item survey administered to participants aged ≥60 years, the interview was structured in three sequential blocks corresponding to the sociodemographic questionnaire, the OHAS, and the PSAS; brief rest pauses were offered between blocks, and the researcher verbally confirmed participant comfort before proceeding. With regard to data security, all responses entered into the Google Forms platform were linked to anonymized participant codes rather than identifiable information; access to the form was restricted to the research team via password-protected institutional accounts, and the dataset was exported to an encrypted local file immediately following completion of data collection.

### 2.5 Statistical analysis

Data were analyzed using IBM SPSS Statistics, version 26 (IBM Corp). Normality was assessed using Kolmogorov–Smirnov and Shapiro–Wilk tests; non-normal distributions were identified, warranting the use of nonparametric methods for group comparisons and bivariate analyses. Between-group comparisons were conducted using the Mann–Whitney U test (2 groups) and Kruskal-Wallis test (≥3 groups), with Bonferroni-corrected Dunn *post hoc* tests applied for pairwise comparisons. Bivariate associations were assessed using Spearman rank correlation coefficients. Multivariate linear regression (Enter method, 8 simultaneous predictors: age, gender, lifestyle [urban/rural], prior removable denture use, opinion about implant, psychiatric medication use, education level, and number of implants) was conducted separately for OHAS (total score as the dependent variable) and for each PSAS subscale (Enjoyment, Perceived Importance, and Anxiety analyzed as separate dependent variables). Although the raw scale scores showed non-normal distributions, the use of multivariate linear regression is methodologically justified for several reasons. First, linear regression is considered robust to moderate departures from normality when sample sizes are sufficient, particularly for the total sample (*N* = 277), where central limit theorem considerations apply. Second, linear regression was employed for multivariate adjustment because no nonparametric equivalent is available for simultaneous control of multiple covariates. Third, the continuous and ordinal nature of the dependent variables (OHAS total score and PSAS subscales) satisfies the measurement-level requirement for linear regression. Prior to analysis, regression assumptions were evaluated: residual normality was assessed using P–P plots and the Kolmogorov–Smirnov test; homoscedasticity was evaluated by inspection of scatterplots of standardized residuals against predicted values; multicollinearity was examined via variance inflation factors (all VIF < 2.5); and influential cases were identified using Cook’s distance (threshold: 4/N). No assumption violations that would invalidate the regression models were detected. Throughout this paper, the term “predictor” refers to the independent variables entered into the regression models in a purely statistical sense and does not imply causal or temporal priority, consistent with the cross-sectional study design. Given the exploratory nature of this first study in septuagenarians, no correction for multiple comparisons (e.g., Bonferroni) was applied to the regression analyses; applying such corrections would substantially increase Type II error risk for novel findings. This constitutes a limitation, and future confirmatory studies should employ pre-registered significance thresholds with appropriate alpha adjustment. The significance threshold was set at *p* < 0.05 for all analyses.

### 2.6 Ethical considerations

The study was approved by the Inonu University Health Sciences Scientific Research Ethics Committee (Decision No. 2025/8216; September 16, 2025) and conducted in accordance with the Declaration of Helsinki. Written informed consent was obtained from all participants prior to data collection. Of the 277 participants, 53 (19.1%) were illiterate, including 22 (25.9%) of the 85 septuagenarian participants. For illiterate participants, the study purpose and procedures were explained verbally in full; consent was obtained via witnessed thumbprint signature on the consent form, in the presence of a literate witness who co-signed the document. This procedure was approved as part of the ethics committee decision. Data were stored securely and anonymized prior to analysis.

## 3 Results

### 3.1 Participant characteristics

Of the 277 participants, 85 (30.7%) were aged ≥70 years (septuagenarian subgroup) and 192 (69.3%) were aged 60–69 years. In the septuagenarian subgroup, mean age was 74.6 (SD 5.1) years (range 70–97); 45 (52.9%) were male and 40 (47.1%) were female. The majority of participants resided in urban areas (58.8%), and 63.5% reported prior removable denture use. In the total sample, mean OHAS score was 144.2 (SD 28.2; range 61–205); 138 participants (49.8%) were classified in the OHAS-high category. PSAS subscale means were: Enjoyment 24.3 (SD 7.9; range 8–40), Perceived Importance 17.0 (SD 4.7; range 5–25), and Anxiety 8.4 (SD 3.3; range 3–15; higher score = lower anxiety). In the septuagenarian subgroup, mean OHAS was 141.2 (SD 29.5); PSAS subscale means were Enjoyment 23.2 (SD 8.8), Perceived Importance 16.4 (SD 4.7), and Anxiety 8.3 (SD 3.3). No significant differences between age groups were observed for OHAS or any PSAS subscale (all *p* > 0.05). Significant positive correlations were found between OHAS and PSAS-Enjoyment (*ρ* = 0.55, *p* < 0.001) and PSAS-Perceived Importance (*ρ* = 0.50, *p* < 0.001), and a significant negative correlation with PSAS-Anxiety (*ρ* = −0.39, p < 0.001) in the total sample; these associations were stronger in the septuagenarian subgroup (*ρ* = 0.63, 0.59, and −0.45, respectively, all *p* < 0.001). Sociodemographic characteristics and descriptive statistics for both groups are summarized in Table 1.

**Table 1:** Sociodemographic characteristics and OHAS/PSAS scale scores—Total Sample (*N* = 277).

Sociodemographic status			Sensitivity	Importance	Avoidance	Tendency	Awareness	S. influence	OHAS	Enjoyment	P. importance	Anxiety	PSAS (total)
Features		*N* (%)	42.9 ± 8.6 (21–60)	21.5 ± 4.5 (10–30)	24.7 ± 4.9 (11–35)	20.6 ± 4.9 (6–30)	21.0 ± 4.4 (7–30)	13.6 ± 3.5 (4–20)	144.2 ± 28.2 (61–205)	24.3 ± 7.9 (8–40)	17.0 ± 4.7 (10–30)	8.4 ± 3.3 (3–15)	49.6 ± 10.7 (26–80)
Total		277 (100%)	44/36	22/24	25/27	21/20	21/22	14/14	138 (49.8%)	24/24	18/20	8/6	50/51
Age group	Septuagenarian	85 (30.7%)	*Z* = −1.4 *p* = 0.2 41.7 ± 0.9	*Z* = −0.8 *p* = 0.5 21.0 ± 0.5	*Z* = −1.1 *p* = 0.2 24.1 ± 0.5	*Z* = −0.3 *p* = 0.8 20.5 ± 0.5	*Z* = −1.0 *p* = 0.3 20.4 ± 0.5	*Z* = −0.8 *p* = 0.4 13.3 ± 0.4	*X* = 1.3 *p* = 0.2 100 (52.1%)	*Z* = −1.2 *p* = 0.2 23.2 ± 0.9	*Z* = −1.3 *p* = 0.2 16.4 ± 0.6	*Z* = −0.5 *p* = 0.6 8.3 ± 0.4	*Z* = −1.5 *p* = 0.1 47.9 ± 1.2
	Other	192 (69.3%)	43.4 ± 0.6	21.6 ± 0.3	24.8 ± 0.4	20.6 ± 0.4	21.2 ± 0.3	13.7 ± 0.3	38 (44.7%)	24.8 ± 0.5	17.2 ± 0.3	8.4 ± 0.2	50.4 ± 0.7
Age	Mean ± SD	67.53 ± 5.9 (60–97)	S.rho = −0.08 *p* = 0.2	S.rho = −0.06 *p* = 0.3	S.rho = −0.06 *p* = 0.3	S.rho = −0.25 *p* = 0.7	S.rho = −0.08 *p* = 0.2	S.rho = −0.08 *p* = 0.2	*Z* = −1.6 *p* = 0.1	S.rho = −0.08 *p* = 0.2	S.rho = −0.08 *p* = 0.2	S.rho = −0.02 *p* = 0.8	S.rho = −0.09 *p* = 0.1
	Median/mode	67/60											
Gender	Male	152 (54.9%)	*Z* = −0.7 *p* = 0.5 42.6 ± 0.7	*Z* = −0.4 *p* = 0.6 21.73 ± 0.4	*Z* = −1.3 *p* = 0.2 24.2 ± 0.4	*Z* = −0.6 *p* = 0.5 20.3 ± 0.4	*Z* = −0.7 *p* = 0.4 20.8 ± 0.4	*Z* = −0.5 *p* = 0.5 13.5 ± 0.3	*X* = 0.8 *p* = 0.2 72 (47.4%)	*Z* = −0.5 *p* = 0.6 24.5 ± 0.6	*Z* = −0.6 *p* = 0.5 17.2 ± 0.4	*Z* = −1.2 *p* = 0.2 8.1 ± 0.3	*Z* = −0.5 *p* = 0.6 49.9 ± 0.8
	Female	125 (45.1%)	43.4 ± 0.7	21.6 ± 0.4	25.1 ± 0.4	20.8 ± 0.4	21.2 ± 0.4	13.8 ± 0.3	55 (52.8%)	24.0 ± 0.7	16.7 ± 0.4	8.6 ± 0.3	49.3 ± 0.9
Education Level	Illiterate	53 (19.1%)	H = 34.4 *p* < 0.001 37.2 ± 0.9	H = 35.0 *p* < 0.001 18.6 ± 0.5	H = 26.8 *p* < 0.001 22.1 ± 0.6	H = 10.7 *p* = 0.06 19.4 ± 0.6	H = 23.6 *p* < 0.001 19.2 ± 0.5	H = 36.0 *p* < 0.001 11.2 ± 0.4	*X* = 26.1 *p* = 0.9 33 (47.8%)	H = 47.3 *p* < 0.001 18.7 ± 0.9	H = 19.1 *p* = 0.002 15.9 ± 0.5	H = 1.7 *p* = 0.9 8.1 ± 0.4	H = 43.9 *p* < 0.001 42.7 ± 1.2
	Literate	26 (9.4%)	42.2 ± 1.7	21.0 ± 0.9	24.6 ± 0.9	20.4 ± 0.9	20.5 ± 0.9	13.6 ± 0.7	35 (68.6%)	23.7 ± 1.4	15.4 ± 0.9	8.5 ± 0.6	47.7 ± 1.9
	Primary	69 (24.9%)	44.3 ± 0.9	21.6 ± 0.5	24.9 ± 0.6	20.3 ± 0.6	20.6 ± 0.5	13.7 ± 0.4	10 (38.5%)	24.1 ± 0.9	16.5 ± 0.5	8.6 ± 0.4	49.2 ± 1.2
	Secondary	49 (17.7%)	43.5 ± 1.1	20.8 ± 0.4	24.7 ± 0.7	20.3 ± 0.7	20.9 ± 0.7	14.6 ± 0.4	13 (24.5%)	24.9 ± 0.9	17.0 ± 0.6	8.6 ± 0.5	50.6 ± 1.3
	Highschool	51 (18.4%)	45.6 ± 1.1	23.3 ± 0.6	26.4 ± 0.7	21.5 ± 0.7	22.7 ± 0.6	14.2 ± 0.5	28 (57.1%)	27.2 ± 1.0	18.4 ± 0.6	8.2 ± 0.5	53.9 ± 1.3
	University	29 (10.5%)	44.9 ± 1.9	22.6 ± 0.9	26.0 ± 1.1	22.3 ± 0.9	22.3 ± 0.9	15.1 ± 0.1	19 (65.5%)	29.3 ± 1.6	18.7 ± 1.0	7.9 ± 0.6	55.9 ± 2.3
Marital Status	Single	61 (22.0%)	*Z* = −0.6 *p* = 0.5 42.1 ± 9.4	*Z* = −0.9 *p* = 0.4 20.9 ± 4.9	*Z* = −0.2 *p* = 0.9 24.5 ± 5.4	*Z* = −0.1 *p* = 0.9 20.6 ± 4.5	*Z* = −0.6 *p* = 0.6 20.5 ± 4.5	*Z* = −0.8 *p* = 0.4 13.1 ± 3.5	*X* = 0.2 *p* = 0.4 32 (52.5%)	*Z* = −0.4 *p* = 0.6 24.0 ± 7.9	*Z* = −1.5 *p* = 0.1 17.8 ± 4.5	*Z* = −1.7 *p* = 0.1 7.8 ± 3.3	*Z* = −0.2 *p* = 0.8 49.6 ± 3.3
	Married	216 (78.0%)	43.1 ± 8.4	21.6 ± 4.4	24.7 ± 4.8	20.6 ± 5.0	21.1 ± 4.4	13.7 ± 3.5	106 (49.1%)	24.4 ± 7.8	16.7 ± 4.8	8.5 ± 3.2	49.6 ± 10.6
Family type	Nuclear	180 (65.0%)	*Z* = −0.7 *p* = 0.5 42.8 ± 8.0	*Z* = −1.3 *p* = 0.2 21.2 ± 4.2	*Z* = −0.8 *p* = 0.4 24.5 ± 4.6	*Z* = −1.5 *p* = 0.1 20.3 ± 4.7	*Z* = −1.3 *p* = 0.2 20.8 ± 4.1	*Z* = −1.5 *p* = 0.1 13.4 ± 3.4	*X* = 3.7 *p* = 0.04 82 (45.6%)	*Z* = −1.5 *p* = 0.1 24.9 ± 7.3	*Z* = −1.5 *p* = 0.1 17.2 ± 4.5	*Z* = −0.1 *p* = 0.3 8.2 ± 3.2	*Z* = −1.3 *p* = 0.2 50.2 ± 9.9
	Extended	97 (35.0%)	42.2 ± 9.6	21.9 ± 5.0	24.9 ± 5.5	21.1 ± 5.1	21.3 ± 4.9	14.0 ± 3.6	56 (57.7%)	23.3 ± 8.7	16.5 ± 5.0	8.6 ± 3.2	48.5 ± 11.9
Number of children alive	Mean ± SD	3.1 ± 2.4 (0–18)	S.rho = 0.06 *p* = 0.3	S.rho = 0.07 *p* = 0.2	S.rho = 0.03 *p* = 0.7	S.rho = −0.05 *p* = 0.5	S.rho = 0.004 *p* = 0.9	S.rho = 0.07 *p* = 0.3	*Z* = −0.2 *p* = 0.9	S.rho = 0.06 *p* = 0.9	S.rho = −0.02 *p* = 0.7	S.rho = 0.07 *p* = 0.2	S.rho = 0.01 *p* = 0.9
	Median/mode	3/3											
How many people live in the house	Mean ± SD	3.26 ± 2.3 (1–23)	S.rho = −0.07 *p* = 0.3	S.rho = −0.05 *p* = 0.4	S.rho = −0.08 *p* = 0.2	S.rho = −0.01 *p* = 0.8	S.rho = −0.05 *p* = 0.4	S.rho = −0.04 *p* = 0.5	*Z* = 1.2 *p* = 0.2	S.rho = −0.04 *p* = 0.5	S.rho = −0.06 *p* = 0.3	S.rho = 0.06 *p* = 0.3	S.rho = −0.05 *p* = 0.4
	Median/mode	3/2											
House type	Apartment	136 (49.1%)	*Z* = −1.4 *p* = 0.1 43.6 ± 8.2	*Z* = −1.4 *p* = 0.1 21.8 ± 4.3	*Z* = −1.4 *p* = 0.2 25.0 ± 4.9	*Z* = −0.5 *p* = 0.6 20.7 ± 4.9	*Z* = −0.6 *p* = 0.5 21.1 ± 4.4	*Z* = −0.4 *p* = 0.6 13.7 ± 3.5	*X* = 1.0 *p* = 0.2 72 (52.9%)	*Z* = −1.7 *p* = 0.09 25.1 ± 7.4	*Z* = −1.4 *p* = 0.1 17.4 ± 4.5	*Z* = −0.8 *p* = 0.3 8.5 ± 3.3	*Z* = −2.2 *p* = 0.02 51.0 ± 10.3
	Detached	141 (50.9%)	42.3 ± 8.9	21.0 ± 4.7	24.3 ± 5.0	20.5 ± 4.8	20.8 ± 4.4	13.5 ± 3.5	66 (46.8%)	23.6 ± 8.2	16.6 ± 4.9	7.2 ± 3.2	48.3 ± 10.9
Lifestyle	Urban	182 (65.7%)	*Z* = −3.0 *p* < 0.001 44.2 ± 8.4	*Z* = −3.0 *p* < 0.001 22.1 ± 4.4	*Z* = −4.2 *p* < 0.001 25.5 ± 4.9	*Z* = −3.2 *p* = 0.001 21.2 ± 4.8	*Z* = −3.2 *p* = 0.001 21.5 ± 4.5	*Z* = −3.0 *p* = 0.003 14.1 ± 3.4	*X* = 17.1 *p* < 0.001 107 (58.8%)	*Z* = −3.2 *p* = 0.001 25.4 ± 7.8	*Z* = −1.8 *p* = 0.07 17.3 ± 4.6	*Z* = −1.5 *p* = 0.1 8.1 ± 3.2	*Z* = −2.8 *p* = 0.006 50.9 ± 10.5
	Rural	95 (34.3%)	40.4 ± 8.5	20.1 ± 4.4	23.1 ± 4.6	19.3 ± 4.7	20.0 ± 4.1	12.7 ± 3.6	31 (22.6%)	22.2 ± 7.6	16.3 ± 4.8	8.7 ± 3.2	47.3 ± 10.7
Time with friends or family	0–2	86 (31.0%)	H = 7.2 *p* = 0.03 41.3 ± 9.0	H = 12.9 *p* = 0.002 20.3 ± 4.7	H = 7.0 *p* = 0.03 23.8 ± 5.1	H = 7.2 *p* = 0.03 19.5 ± 4.9	H = 10.7 *p* = 0.005 19.8 ± 4.7	H = 7.4 *p* = 0.02 12.7 ± 3.7	*X* = 9.2 *p* = 0.003 35 (40.7%)	H = 11.5 *p* = 0.003 22.1 ± 8.3	H = 12.7 *p* = 0.002 16.1 ± 5.0	H = 3.1 *p* = 0.2 8.8 ± 3.4	H = 13.8 *p* = 0.001 47.0 ± 11.2
	3–4	125 (45.1%)	42.8 ± 8.6	21.4 ± 4.5	24.6 ± 4.9	20.9 ± 4.6	21.1 ± 4.0	13.7 ± 3.2	60 (48.0%)	24.6 ± 7.4	16.7 ± 4.5	7.9 ± 2.9	49.2 ± 9.9
	5+	66 (23.9%)	45.2 ± 7.6	23.1 ± 3.8	26.0 ± 4.6	21.4 ± 5.2	22.1 ± 4.4	14.4 ± 3.5	43 (65.2%)	26.5 ± 7.4	18.6 ± 4.1	8.6 ± 3.5	53.8 ± 10.3
Number of implants	Mean ± SD	4.38 ± 3.1 (1–16)	S.rho = 0.1 *p* = 0.08	S.rho = 0.1 *p* = 0.05	S.rho = 0.3 *p* = 0.5	S.rho = −0.3 *p* = 0.5	S.rho = 0.5 *p* = 0.4	S.rho = 0.02 *p* = 0.7	*Z* = −0.8 *p* = 0.4	S.rho = 0.03 *p* = 0.6	S.rho = −0.07 *p* = 0.3	S.rho = 0.05 *p* = 0.3	S.rho = 0.02 *p* = 0.8
	Median/mode	3/2											
implant treatment (year)	Mean ± SD	2.68 ± 1.93 (1–12)	S.rho = −0.03 *p* = 0.6	S.rho = −0.08 *p* = 0.2	S.rho = −0.02 *p* = 0.7	S.rho = 0.03 *p* = 0.6	S.rho = −0.04 *p* = 0.5	S.rho = −0.05 *p* = 0.4	*Z* = 1.3 *p* = 0.2	S.rho = −0.12 *p* = 0.05	S.rho = −0.08 *p* = 0.2	S.rho = −0.10 *p* = 0.1	S.rho = −0.15 *p* = 0.01
	Median/mode	2/1											
Prosthetic use before implant	Yes	141 (50.9%)	*Z* = −5.1 *p* < 0.001 40.5 ± 8.1	*Z* = −5.1 *p* < 0.001 20.2 ± 4.2	*Z* = −4.8 *p* < 0.001 23.5 ± 4.9	*Z* = −5.1 *p* < 0.001 19.2 ± 4.8	*Z* = −4.9 *p* < 0.001 19.8 ± 4.1	*Z* = −5.4 *p* < 0.001 12.6 ± 3.3	*X* = 34.0 *p* < 0.001 46 (32.6%)	*Z* = −4.1 *p* < 0.001 22.4 ± 7.5	*Z* = −3.1 *p* < 0.001 16.1 ± 4.7	*Z* = 1.5 *p* = 0.1 8.6 ± 3.3	*Z* = −4.1 *p* < 0.001 47.1 ± 10.3
	No	136 (49.1%)	45.4 ± 8.4	22.8 ± 4.4	26.0 ± 4.6	22.0 ± 4.5	22.2 ± 4.4	14.7 ± 3.4	92 (67.6%)	26.3 ± 7.6	17.8 ± 4.5	8.1 ± 3.2	52.2 ± 10.4
Opinion about implant	Positive	228 (82.3%)	*Z* = −4.5 *p* < 0.001 43.9 ± 8.4	*Z* = −4.4 *p* < 0.001 21.9 ± 4.4	*Z* = −4.3 *p* < 0.001 25.2 ± 5.0	*Z* = −3.0 *p* = 0.003 20.9 ± 5.0	*Z* = −4.2 *p* < 0.001 21.4 ± 4.4	*Z* = −4.2 *p* < 0.001 14.0 ± 3.5	*X* = 20.6 *p* < 0.001 128 (56.1%)	*Z* = −3.8 *p* < 0.001 25.2 ± 7.6	*Z* = −2.4 *p* = 0.02 17.3 ± 4.6	*Z* = −0.8 *p* = 0.4 8.3 ± 3.2	*Z* = −3.7 *p* < 0.001 50.7 ± 10.4
	Negative	49 (17.7%)	38.3 ± 8.1	19.0 ± 4.3	22.2 ± 4.0	19.1 ± 4.0	18.8 ± 3.7	11.8 ± 3.2	10 (20.4%)	20.3 ± 7.8	15.4 ± 5.0	8.6 ± 3.4	44.4 ± 10.4
Psychiatric medication	Yes	56 (20.2%)	*Z* = −3.8 *p* < 0.001 37.7 ± 9.5	*Z* = −3.9 *p* < 0.001 19.2 ± 4.9	*Z* = −3.5 *p* = 0.001 22.5 ± 5.6	*Z* = −2.4 *p* = 0.02 19.3 ± 4.9	*Z* = −2.5 *p* = 0.01 19.5 ± 4.9	*Z* = −2.7 *p* = 0.008 12.3 ± 3.9	*X* = 4.3 *p* = 0.03 21 (37.5%)	*Z* = −3.7 *p* < 0.001 20.7 ± 8.0	*Z* = −3.0 *p* = 0.003 15.3 ± 4.9	*Z* = −1.3 *p* = 0.2 8.8 ± 3.1	*Z* = −3.9 *p* < 0.001 44.8 ± 10.5
	No	221 (79.8%)	44.0 ± 8.0	22.0 ± 4.3	25.2 ± 4.6	20.9 ± 4.8	21.3 ± 4.2	13.9 ± 3.3	117 (52.9%)	25.2 ± 7.6	17.4 ± 4.5	8.2 ± 3.2	50.8 ± 10.4
Chronic disease	No	180 (65.0%)	*Z* = −1.4 *p* = 0.2 42.3 ± 9.4	*Z* = −2.1 *p* = 0.04 21.0 ± 4.8	*Z* = −0.4 *p* = 0.7 24.5 ± 5.3	*Z* = −1.2 *p* = 0.2 20.8 ± 5.0	*Z* = −0.4 *p* = 0.7 21.0 ± 4.7	*Z* = −0.6 *p* = 0.6 13.5 ± 3.6	*X* = 0.5 *p* = 0.3 87 (48.3%)	*Z* = −1.2 *p* = 0.2 23.9 ± 8.3	*Z* = −0.6 *p* = 0.6 17.0 ± 4.9	*Z* = −1.7 *p* = 0.09 8.1 ± 3.3	*Z* = −1.2 *p* = 0.3 49.0 ± 11.0
	Yes	97 (35.0%)	44.0 ± 6.7	22.2 ± 3.7	25.0 ± 4.0	20.0 ± 4.8	20.9 ± 3.6	13.8 ± 3.2	51 (52.6%)	25.1 ± 7.0	16.8 ± 4.3	8.8 ± 3.2	50.7 ± 10.0
	*Mean ± SD*	1.97 ± 0.99 (1–5)	S.rho = −0.08 *p* = 0.2	S.rho = −0.12 *p* = 0.04	S.rho = −0.02 *p* = 0.7	S.rho = 0.07 *p* = 0.2	S.rho = 0.2 *p* = 0.7	S.rho = −0.03 *p* = 0.5	*Z* = 2.0 *p* = 0.03	S.rho = −0.07 *p* = 0.2	S.rho = −0.03 *p* = 0.5	S.rho = −0.10 *p* = 0.09	S.rho = −0.07 *p* = 0.2
	Median/Mode	2/1											
Health perception	Good	137 (49.5%)	*Z* = −2.4 *p* = 0.02 44.1 ± 8.6	*Z* = −1.8 *p* = 0.07 22.0 ± 4.5	*Z* = −2.3 *p* = 0.02 25.3 ± 5.0	*Z* = −1.8 *p* = 0.08 21.0 ± 4.9	*Z* = −2.3 *p* = 0.03 21.5 ± 4.5	*Z* = −2.0 *p* = 0.04 14.0 ± 3.5	*X* = 5.5 *p* = 0.01 78 (56.9%)	*Z* = −1.5 *p* = 0.2 25.0 ± 8.0	*Z* = −2.0 *p* = 0.04 17.5 ± 4.8	*Z* = −0.3 *p* = 0.8 8.4 ± 3.2	*Z* = −1.9 *p* = 0.06 51.0 ± 11.1
	Bad and Mid	140 (50.5%)	41.8 ± 8.5	21.0 ± 4.5	24.0 ± 4.0	20.0 ± 4.8	20.3 ± 4.2	13.2 ± 3.5	60 (42.9%)	23.0 ± 7.5	16.5 ± 4.5	8.3 ± 3.3	48.3 ± 10.1
Income	below minimum wage	182 (65.7%)	*Z* = −1.0 *p* = 0.3 42.5 ± 8.5	*Z* = −1.7 *p* = 0.1 21.1 ± 4.6	*Z* = −0.5 *p* = 0.6 24.6 ± 4.8	*Z* = −0.3 *p* = 0.8 20.6 ± 4.7	*Z* = −0.9 *p* = 0.4 20.8 ± 4.4	*Z* = −0.6 *p* = 0.6 13.5 ± 3.5	*X* = 0.9 *p* = 0.2 87 (47.8%)	*Z* = −0.4 *p* = 0.7 24.2 ± 7.8	*Z* = −0.8 *p* = 0.5 16.8 ± 4.6	*Z* = −2.6 *p* = 0.008 8.0 ± 3.2	*Z* = −1.3 *p* = 0.2 48.9 ± 10.3
	above minimum wage	95 (34.3%)	43.6 ± 8.7	22.0 ± 4.4	24.8 ± 5.1	20.4 ± 5.2	21.2 ± 4.5	13.8 ± 3.5	51 (53.7%)	24.6 ± 7.9	17.2 ± 4.8	9.0 ± 3.2	50.9 ± 11.2
Places	Malatya	256 (92.4%)	*Z* = −2.5 *p* = 0.01 42.5 ± 8.6	*Z* = −1.8 *p* = 0.07 21.3 ± 4.5	*Z* = −2.8 *p* = 0.005 24.4 ± 5.0	*Z* = −1.5 *p* = 0.1 20.4 ± 5.0	*Z* = −2.2 *p* = 0.03 20.8 ± 4.4	*Z* = −2.4 *p* = 0.02 13.4 ± 3.5	*X* = 4.2 *p* = 0.03 123 (48.0%)	*Z* = −1.9 *p* = 0.05 24.0 ± 7.8	*Z* = −1.9 *p* = 0.7 16.8 ± 4.7	*Z* = −0.4 *p* = 0.7 8.4 ± 3.2	*Z* = −1.9 *p* = 0.06 49.2 ± 10.5
	Other	21 (7.6%)	47.5 ± 7.3	23.3 ± 3.5	27.6 ± 4.0	22.2 ± 4.2	23.0 ± 3.5	15.4 ± 2.3	15 (71.4%)	27.6 ± 7.5	19.0 ± 3.5	8.1 ± 3.0	54.6 ± 11.3
Correlations between scales									S.rho = 0.48 *p* < 0.001 *p* < 0.001				Enjoyment: *ρ* = 0.55, *p* < 0.001 P. importance: *ρ* = 0.50, *p* < 0.001 Anxiety: *ρ* = −0.39, *p* < 0.001 *p* < 0.001

### 3.2 Group comparisons by sociodemographic variables

In the septuagenarian subgroup, several sociodemographic variables were significantly associated with OHAS and PSAS scores. Males scored significantly higher than females on OHAS Sensitivity (*Z* = 2.66, *p* = 0.008), Importance (*Z* = 2.97, *p* = 0.003), PSAS-Enjoyment (*Z* = 2.76, *p* = 0.006), and total OHAS (*Z* = 2.13, *p* = 0.034); however, the proportion in the OHAS-high category was higher in females (67.5%) than males (44.4%). Urban residents scored consistently higher than rural residents across all subscales (all *p* ≤ 0.003), although the OHAS-high proportion was lower in urban participants (38.0% vs. 80.0%). Prior removable denture users scored significantly lower on all OHAS subscales, all PSAS subscales, and total OHAS (all *p* ≤ 0.011); the OHAS-high proportion was 70.4% among prior denture users vs. 29.0% among non-users. Those with a positive opinion about their implant scored higher on all scales, with the largest effects on PSAS-Enjoyment (*Z* = 4.20, *p* < 0.001) and total OHAS (*Z* = 3.88, *p* < 0.001). Group comparisons are presented in Table 2.

**Table 2:** Education level—post−hoc pairwise comparisons, total sample (Dunn test, Bonferroni correction).

Scale	Comparison	*p* (Bonferroni)
Sensitivity	Highschool vs. Illiterate	*p* < 0.001
	Primary vs. Illiterate	*p* < 0.001
	Illiterate vs. University	*p* = 0.001
	Illiterate vs. Secondary	*p* = 0.004
Importance	Highschool vs. Illiterate	*p* < 0.001
	Illiterate vs. University	*p* = 0.002
	Illiterate vs. Secondary	*p* = 0.003
	Primary vs. Illiterate	*p* = 0.003
Avoidance	Highschool vs. Illiterate	*p* < 0.001
	Illiterate vs. University	*p* = 0.004
	Primary vs. Illiterate	*p* = 0.02
Awareness	Highschool vs. Illiterate	*p* < 0.001
	Illiterate vs. University	*p* = 0.01
S. influence	Illiterate vs. Secondary	*p* < 0.001
	Illiterate vs. University	*p* < 0.001
	Highschool vs. Illiterate	*p* < 0.001
	Primary vs. Illiterate	*p* = 0.002
Enjoyment	Illiterate vs. University	*p* < 0.001
	Highschool vs. Illiterate	*p* < 0.001
	Illiterate vs. Secondary	*p* < 0.001
	Primary vs. Illiterate	*p* = 0.001
P. importance	Highschool vs. Illiterate	*p* = 0.02
	Illiterate vs. University	*p* = 0.03
PSAS	Highschool vs. Illiterate	*p* < 0.001
	Illiterate vs. University	*p* < 0.001
	Illiterate vs. Secondary	*p* = 0.001
	Primary vs. Illiterate	*p* = 0.008

Only statistically significant pairs (*p* < 0.05 after Bonferroni correction) are shown.

Education level was significantly associated with OHAS and PSAS scores in both the total sample and the septuagenarian subgroup. In the septuagenarian subgroup, significant differences were observed for OHAS Sensitivity (*H* = 13.38, *p* = 0.020), Importance (*H* = 17.92, *p* = 0.003), Awareness (*H* = 11.66, *p* = 0.040), Social Influence (*H* = 18.70, *p* = 0.002), PSAS-Enjoyment (*H* = 28.18, *p* < 0.001), PSAS (*H* = 25.74, *p* < 0.001), and OHAS category (*H* = 13.34, *p* = 0.020). Bonferroni-corrected Dunn *post hoc* comparisons for the total sample are presented in Table 3.

**Table 3:** Time with friends or family—post-hoc pairwise comparisons, total sample (Dunn test, Bonferroni correction).

Scale	Comparison	*p* (Bonferroni)
Sensitivity	0–2 vs. 5+	*p* = 0.02
Importance	0–2 vs. 5+	*p* = 0.001
	3–4 vs. 5+	*p* = 0.04
Avoidance	0–2 vs. 5+	*p* = 0.03
Tendency	0–2 vs. 5+	*p* = 0.03
Awareness	0–2 vs. 5+	*p* = 0.003
S. influence	0–2 vs. 5+	*p* = 0.02
Enjoyment	0–2 vs. 5+	*p* = 0.003
P. importance	0–2 vs. 5+	*p* = 0.002
	3–4 vs. 5+	*p* = 0.01
PSAS	0–2 vs. 5+	*p* < 0.001
	3–4 vs. 5+	*p* = 0.03

Groups: 0–2 days/week, 3–4 days/week, 5+ days/week. Significant pairs only.

Social interaction frequency was significantly associated with attitude scores in the total sample. Significant group differences were observed for OHAS Importance, Awareness, PSAS-Enjoyment, PSAS-Perceived Importance, and PSAS (all *p* ≤ 0.027); participants socializing 0–2 days per week scored significantly lower than those socializing 3–4 days per week on these five scales (all corrected *p* ≤ 0.029). Pairwise comparisons for the total sample are presented in Tables 2, 3. In the septuagenarian subgroup (see Table 4 for subgroup characteristics), Kruskal–Wallis tests revealed significant group differences for PSAS-Enjoyment (*H* = 11.23, *p* = 0.004), PSAS-Perceived Importance (*H* = 7.21, p = 0.027), and PSAS (*H* = 9.87, *p* = 0.007). Post hoc analyses showed that participants socializing 0–2 days scored significantly lower than those socializing 3–4 days on PSAS-Enjoyment (*Z* = −3.31, p = 0.003), PSAS-Perceived Importance (*Z* = −2.68, p = 0.020), and PSAS (*Z* = −3.14, *p* = 0.005). Pairwise comparisons for the septuagenarian subgroup are presented in Tables 5, 6.

**Table 4:** Sociodemographic characteristics and OHAS/PSAS scale scores—septuagenarian subgroup (*N* = 85).

Sociodemographic Status			Sensitivity	Importance	Avoidance	Tendency	Awareness	S. influence	OHAS	Enjoyment	P. importance	Anxiety	PSAS (total)
Features		85 (100%)	41.7 ± 8.8 (21–60)	21.0 ± 4.8 (10–30)	24.2 ± 5.1 (11–35)	20.5 ± 4.6 (8–30)	20.4 ± 4.8 (7–30)	13.3 ± 3.4 (4–20)	141.2 ± 29.5 (61–205)	23.2 ± 8.8 (8–40)	16.4 ± 4.7 (5–25)	8.3 ± 3.3 (3–15)	47.9 ± 11.4 (26–80)
Total		85 (100%)	42/52	22/24	24/22	21/22	20/20	14/15	47 (55.3%)	24/16	17/20	8/6	49/51
Age	Mean ± SD	74.55 ± 5.12 (70–97)	S.rho = −0.19 *p* = 0.08	S.rho = −0.18 *p* = 0.09	S.rho = −0.20 *p* = 0.07	S.rho = −0.19 *p* = 0.08	S.rho = −0.16 *p* = 0.2	S.rho = −0.16 *p* = 0.1	*Z* = 1.5 *p* = 0.1 75.0 ± 4.9	S.rho = −0.16 *p* = 0.1	S.rho = −0.23 *p* = 0.03	S.rho = 0.06 *p* = 0.6	S.rho = −0.20 *p* = 0.07
	Median/mode	73/70							74.0 ± 5.4				
Gender	Male	45 (52.9%)	*Z* = 2.7 *p* = 0.008 44.0 ± 9.0	*Z* = 3.0 *p* = 0.003 22.4 ± 4.7	*Z* = 1.6 *p* = 0.1 25.0 ± 5.3	*Z* = 1.2 *p* = 0.2 20.9 ± 4.9	*Z* = 1.8 *p* = 0.07 21.3 ± 5.0	*Z* = 1.7 *p* = 0.09 13.8 ± 3.6	*X* = 3.7 *p* = 0.06 20 (44.4%)	*Z* = 2.9 *p* = 0.003 25.8 ± 8.2	*Z* = 1.4 *p* = 0.2 17.0 ± 4.4	*Z* = −0.5 *p* = 0.6 8.1 ± 3.3	*Z* = 2.8 *p* = 0.006 51.0 ± 11.1
	Female	40 (47.1%)	39.2 ± 7.9	19.5 ± 4.5	23.3 ± 4.8	20.1 ± 4.1	19.4 ± 4.5	12.8 ± 3.2	27 (67.5%)	20.3 ± 8.6	15.6 ± 4.9	8.4 ± 3.2	44.4 ± 10.8
Education Level	Illiterate	22 (25.9%)	H = 13.4 *p* = 0.02 37.5 ± 8.0	H = 17.9 *p* = 0.003 18.8 ± 4.0	H = 10.2 *p* = 0.07 22.5 ± 4.3	H = 7.8 *p* = 0.2 20.0 ± 3.5	H = 11.7 *p* = 0.04 19.1 ± 4.1	H = 18.7 *p* = 0.002 11.0 ± 3.1	*X* = 15.7 *p* = 0.008 17 (77.3%)	H = 28.2 *p* < 0.001 16.2 ± 5.5	H = 10.3 *p* = 0.07 15.5 ± 4.5	H = 6.4 *p* = 0.3 8.0 ± 3.2	H = 25.7 *p* < 0.001 39.6 ± 8.1
	Literate	10 (11.8%)	42.7 ± 10.4	21.0 ± 5.7	24.3 ± 6.3	20.2 ± 5.9	19.7 ± 5.9	13.2 ± 4.7	6 (60.0%)	20.6 ± 9.0	14.1 ± 5.2	9.6 ± 3.6	44.3 ± 11.3
	Primary	25 (29.4%)	41.7 ± 7.0	20.5 ± 4.1	23.6 ± 4.9	20.1 ± 4.7	20.1 ± 4.3	13.6 ± 2.5	16 (64.0%)	25.3 ± 7.3	16.0 ± 3.8	8.1 ± 2.7	49.5 ± 9.5
	Secondary	11 (12.9%)	41.7 ± 8.4	21.6 ± 4.0	24.5 ± 4.1	19.4 ± 3.6	19.6 ± 4.8	14.5 ± 2.8	5 (45.5%)	24.5 ± 6.1	17.2 ± 4.2	8.5 ± 3.6	50.2 ± 7.8
	Highschool	14 (16.5%)	47.4 ± 8.3	24.8 ± 4.1	27.3 ± 4.7	23.0 ± 4.6	23.9 ± 4.3	15.6 ± 2.9	2 (14.3%)	30.4 ± 7.6	19.2 ± 4.1	7.1 ± 3.3	56.7 ± 9.0
	University	3 (3.5%)	43.3 ± 16.8	22.7 ± 9.3	24.7 ± 9.5	21.3 ± 8.1	21.7 ± 8.4	13.3 ± 4.7	1 (33.3%)	28.3 ± 17.7	17.3 ± 10.8	11.7 ± 4.9	57.3 ± 27.3
Marital Status	Single	22 (25.9%)	*Z* = 0.5 *p* = 0.6 42.4 ± 8.2	*Z* = 0.5 *p* = 0.6 21.5 ± 4.9	*Z* = 0.7 *p* = 0.5 24.9 ± 4.8	*Z* = 0.1 *p* = 1.0 20.7 ± 3.8	*Z* = 0.4 *p* = 0.7 20.7 ± 5.0	*Z* = 0.6 *p* = 0.5 13.6 ± 3.4	*X* = 0.7 *p* = 0.4 10 (45.5%)	*Z* = −0.2 *p* = 0.9 23.3 ± 8.3	*Z* = 0.3 *p* = 0.8 16.8 ± 4.2	*Z* = −0.7 *p* = 0.5 7.9 ± 3.3	*Z* = −0.1 *p* = 0.9 47.9 ± 9.9
	Married	63 (74.1%)	41.5 ± 9.1	20.9 ± 4.8	23.9 ± 5.2	20.5 ± 4.8	20.3 ± 4.8	13.2 ± 3.5	37 (58.7%)	23.2 ± 9.0	16.2 ± 4.9	8.4 ± 3.3	47.9 ± 12.0
Family Type	Nuclear	55 (64.7%)	*Z* = 0.0 *p* = 1.0 41.7 ± 8.4	*Z* = −0.2 *p* = 0.9 21.0 ± 4.5	*Z* = 0.1 *p* = 0.9 24.2 ± 4.9	*Z* = −0.3 *p* = 0.8 20.5 ± 4.2	*Z* = −0.0 *p* = 1.0 20.4 ± 4.6	*Z* = −0.8 *p* = 0.4 13.1 ± 3.4	*X* = 0.2 *p* = 0.6 32 (58.2%)	*Z* = 1.2 *p* = 0.2 24.1 ± 8.1	*Z* = 0.4 *p* = 0.7 16.7 ± 4.1	*Z* = −1.2 *p* = 0.2 7.9 ± 3.1	*Z* = 1.0 *p* = 0.3 48.8 ± 10.1
	Extended	30 (35.3%)	41.8 ± 9.6	21.2 ± 5.4	24.2 ± 5.5	20.7 ± 5.1	20.5 ± 5.2	13.7 ± 3.4	15 (50.0%)	21.6 ± 9.9	15.7 ± 5.6	8.9 ± 3.5	46.3 ± 13.5
Number of children alive	Mean ± SD	3.15 ± 2.42 (0–12)	S.rho = 0.10 *p* = 0.4	S.rho = 0.08 *p* = 0.5	S.rho = 0.09 *p* = 0.4	S.rho = −0.06 *p* = 0.6	S.rho = 0.05 *p* = 0.6	S.rho = 0.09 *p* = 0.4	*Z* = −0.1 *p* = 0.9 3.2 ± 2.7	S.rho = 0.08 *p* = 0.5	S.rho = −0.05 *p* = 0.7	S.rho = 0.08 *p* = 0.5	S.rho = 0.04 *p* = 0.7
	Median/mode	3/2							3.1 ± 2.1				
How many people live in the house	Mean ± SD	3.22 ± 3.07 (1–23)	S.rho = −0.14 *p* = 0.2	S.rho = −0.20 *p* = 0.07	S.rho = −0.20 *p* = 0.06	S.rho = −0.12 *p* = 0.3	S.rho = −0.15 *p* = 0.2	S.rho = −0.20 *p* = 0.06	*Z* = 1.9 *p* = 0.06 3.7 ± 3.7	S.rho = −0.15 *p* = 0.2	S.rho = −0.17 *p* = 0.1	S.rho = 0.20 *p* = 0.07	S.rho = −0.15 *p* = 0.2
	Median/mode	2/2							2.6 ± 1.8				
House Type	Apartment	37 (43.5%)	*Z* = 1.0 *p* = 0.3 42.4 ± 9.0	*Z* = 1.6 *p* = 0.1 21.8 ± 4.7	*Z* = 1.4 *p* = 0.2 24.8 ± 5.3	*Z* = 0.9 *p* = 0.4 20.9 ± 4.7	*Z* = 1.4 *p* = 0.2 21.1 ± 4.8	*Z* = 0.6 *p* = 0.6 13.4 ± 3.6	*X* = 3.0 *p* = 0.08 16 (43.2%)	*Z* = 1.4 *p* = 0.2 24.7 ± 8.5	*Z* = 0.7 *p* = 0.5 17.0 ± 3.9	*Z* = −0.1 *p* = 0.9 8.4 ± 3.5	*Z* = 1.4 *p* = 0.1 50.1 ± 11.2
	Detached	48 (56.5%)	41.2 ± 8.8	20.4 ± 4.8	23.7 ± 4.9	20.2 ± 4.4	19.9 ± 4.8	13.2 ± 3.3	31 (64.6%)	22.1 ± 8.9	15.9 ± 5.2	8.2 ± 3.1	46.2 ± 11.4
Lifestyle	Urban	50 (58.8%)	*Z* = 3.3 *p* < 0.001 43.9 ± 9.6	*Z* = 3.5 *p* < 0.001 22.4 ± 5.2	*Z* = 3.5 *p* < 0.001 25.6 ± 5.7	*Z* = 3.0 *p* = 0.003 21.6 ± 5.1	*Z* = 3.0 *p* = 0.003 21.4 ± 5.4	*Z* = 3.1 *p* = 0.002 14.2 ± 3.5	*X* = 13.0 *p* < 0.001 19 (38.0%)	*Z* = 3.3 *p* = 0.001 25.8 ± 9.2	*Z* = 1.3 *p* = 0.2 17.0 ± 4.7	*Z* = −0.9 *p* = 0.4 8.1 ± 3.3	*Z* = 3.0 *p* = 0.003 50.9 ± 11.8
	Rural	35 (41.2%)	38.6 ± 6.4	19.2 ± 3.4	22.2 ± 3.2	19.1 ± 3.0	19.0 ± 3.3	12.1 ± 2.9	28 (80.0%)	19.5 ± 6.7	15.5 ± 4.6	8.5 ± 3.2	43.6 ± 9.4
Time with friends or family	0–2	31 (36.5%)	H = 2.8 *p* = 0.3 39.5 ± 9.8	H = 2.7 *p* = 0.3 19.8 ± 5.4	H = 1.6 *p* = 0.5 23.0 ± 5.5	H = 1.2 *p* = 0.5 19.7 ± 5.0	H = 5.7 *p* = 0.06 18.6 ± 5.4	H = 1.7 *p* = 0.4 12.6 ± 4.0	*X* = 0.5 *p* = 0.8 18 (58.1%)	H = 11.2 *p* = 0.004 19.0 ± 8.6	H = 7.2 *p* = 0.03 14.6 ± 4.9	H = 3.9 *p* = 0.1 9.2 ± 3.6	H = 9.9 *p* = 0.007 42.8 ± 11.0
	3–4	39 (45.9%)	43.6 ± 8.3	21.9 ± 4.4	24.9 ± 5.1	20.8 ± 4.6	21.6 ± 4.3	13.9 ± 3.1	20 (51.3%)	26.1 ± 8.1	17.6 ± 4.5	7.6 ± 2.9	51.3 ± 10.5
	5+	15 (17.6%)	41.5 ± 7.1	21.5 ± 4.0	24.7 ± 3.7	21.5 ± 3.2	21.0 ± 4.0	13.1 ± 2.8	9 (60.0%)	24.6 ± 8.1	17.0 ± 3.7	8.1 ± 3.2	49.7 ± 11.3
Number of implants	Mean ± SD	4.28 ± 3.13 (1–16)	S.rho = 0.24 *p* = 0.03	S.rho = 0.27 *p* = 0.01	S.rho = 0.17 *p* = 0.1	S.rho = 0.04 *p* = 0.7	S.rho = 0.17 *p* = 0.1	S.rho = 0.16 *p* = 0.1	*Z* = −2.0 *p* = 0.04 3.6 ± 2.3	S.rho = 0.25 *p* = 0.02	S.rho = 0.05 *p* = 0.7	S.rho = −0.13 *p* = 0.2	S.rho = 0.20 *p* = 0.06
	Median/mode	3/2							5.2 ± 3.8				
implant treatment (year)	Mean ± SD	3.66 ± 2.24 (1–12)	S.rho = −0.03 *p* = 0.8	S.rho = −0.10 *p* = 0.4	S.rho = −0.06 *p* = 0.6	S.rho = 0.05 *p* = 0.7	S.rho = 0.03 *p* = 0.8	S.rho = −0.13 *p* = 0.2	*Z* = 1.1 *p* = 0.3 3.8 ± 2.1	S.rho = −0.15 *p* = 0.2	S.rho = −0.11 *p* = 0.3	S.rho = −0.01 *p* = 1.0	S.rho = −0.18 *p* = 0.1
	Median/mode	3/1							3.5 ± 2.5				
Prosthetic use before implant	Yes	54 (63.5%)	*Z* = −3.3 *p* = 0.001 39.5 ± 8.8	*Z* = −3.3 *p* = 0.001 19.8 ± 4.7	*Z* = −3.3 *p* < 0.001 22.9 ± 5.1	*Z* = −3.2 *p* = 0.001 19.4 ± 4.6	*Z* = −3.0 *p* = 0.003 19.3 ± 4.7	*Z* = −3.2 *p* = 0.001 12.5 ± 3.5	*X* = 12.0 *p* < 0.001 38 (70.4%)	*Z* = −2.5 *p* = 0.01 21.4 ± 8.4	*Z* = −2.3 *p* = 0.02 15.4 ± 4.7	*Z* = 1.2 *p* = 0.2 8.6 ± 3.3	*Z* = −2.6 *p* = 0.009 45.4 ± 10.8
	No	31 (36.5%)	45.6 ± 7.6	23.2 ± 4.3	26.5 ± 4.1	22.5 ± 3.7	22.4 ± 4.4	14.8 ± 2.8	9 (29.0%)	26.5 ± 8.7	18.0 ± 4.2	7.7 ± 3.2	52.3 ± 11.3
Opinion about implant	Positive	64 (75.3%)	*Z* = 3.5 *p* < 0.001 43.5 ± 8.7	*Z* = 2.8 *p* = 0.004 21.8 ± 4.7	*Z* = 3.1 *p* = 0.002 25.0 ± 5.2	*Z* = 1.5 *p* = 0.1 20.9 ± 4.8	*Z* = 3.0 *p* = 0.002 21.2 ± 4.8	*Z* = 3.5 *p* < 0.001 14.0 ± 3.3	*X* = 8.9 *p* = 0.003 29 (45.3%)	*Z* = 4.2 *p* < 0.001 25.5 ± 8.4	*Z* = 2.3 *p* = 0.02 17.1 ± 4.4	*Z* = −1.3 *p* = 0.2 8.0 ± 3.2	*Z* = 3.9 *p* < 0.001 50.6 ± 10.8
	Negative	21 (24.7%)	36.5 ± 7.1	18.7 ± 4.1	21.7 ± 3.7	19.5 ± 3.6	18.1 ± 4.0	11.1 ± 3.0	18 (85.7%)	16.3 ± 5.9	14.1 ± 5.1	9.0 ± 3.4	39.5 ± 9.1
Psychiatric medication	Yes	23 (27.1%)	*Z* = −2.0 *p* = 0.04 38.1 ± 11.6	*Z* = −2.4 *p* = 0.01 18.6 ± 5.8	*Z* = −2.1 *p* = 0.04 22.2 ± 6.6	*Z* = −1.0 *p* = 0.3 19.6 ± 5.8	*Z* = −1.5 *p* = 0.1 19.0 ± 5.8	*Z* = −2.6 *p* = 0.01 11.5 ± 4.5	*X* = 0.8 *p* = 0.4 15 (65.2%)	*Z* = −2.7 *p* = 0.007 19.0 ± 7.4	*Z* = −1.3 *p* = 0.2 15.2 ± 4.6	*Z* = 0.3 *p* = 0.8 8.4 ± 3.3	*Z* = −2.7 *p* = 0.007 42.5 ± 9.5
	No	62 (72.9%)	43.1 ± 7.2	22.0 ± 4.0	24.9 ± 4.2	20.9 ± 4.0	20.9 ± 4.3	14.0 ± 2.7	32 (51.6%)	24.8 ± 8.8	16.8 ± 4.7	8.2 ± 3.3	49.9 ± 11.5
Chronic disease	No	27 (31.8%)	*Z* = 0.2 *p* = 0.8 42.4 ± 5.9	*Z* = 0.3 *p* = 0.8 21.5 ± 3.7	*Z* = −0.4 *p* = 0.7 24.0 ± 3.5	*Z* = −1.5 *p* = 0.1 19.7 ± 3.7	*Z* = −0.6 *p* = 0.6 20.0 ± 4.1	*Z* = 0.1 *p* = 0.9 13.6 ± 2.6	*X* = 0.1 *p* = 0.8 16 (59.3%)	*Z* = 0.3 *p* = 0.7 23.7 ± 7.2	*Z* = −2.5 *p* = 0.01 15.1 ± 3.4	*Z* = 1.0 *p* = 0.3 8.7 ± 3.1	*Z* = −0.2 *p* = 0.8 47.6 ± 9.5
	Yes	58 (68.2%)	41.4 ± 9.9	20.8 ± 5.2	24.2 ± 5.7	20.9 ± 4.9	20.6 ± 5.1	13.2 ± 3.8	31 (53.4%)	23.0 ± 9.5	17.0 ± 5.1	8.0 ± 3.4	48.0 ± 12.3
	Mean ± SD	2.15 ± 1.03 (1–4)	S.rho = −0.10 *p* = 0.6	S.rho = −0.07 *p* = 0.7	S.rho = −0.18 *p* = 0.4	S.rho = −0.05 *p* = 0.8	S.rho = −0.04 *p* = 0.8	S.rho = −0.28 *p* = 0.2	*Z* = 0.6 *p* = 0.6 2.2 ± 1.1	S.rho = −0.45 *p* = 0.02	S.rho = −0.39 *p* = 0.04	S.rho = −0.11 *p* = 0.6	S.rho = −0.54 *p* = 0.004
	Median/mode	2/1							2.0 ± 1.0				
Health perception	Good	50 (58.8%)	*Z* = −1.1 *p* = 0.3 41.0 ± 8.3	*Z* = −1.2 *p* = 0.2 20.5 ± 4.5	*Z* = −1.3 *p* = 0.2 23.6 ± 4.7	*Z* = −1.1 *p* = 0.3 20.2 ± 4.2	*Z* = −1.5 *p* = 0.1 19.8 ± 4.6	*Z* = −1.2 *p* = 0.2 13.0 ± 3.2	*X* = 1.6 *p* = 0.2 31 (62.0%)	*Z* = 0.1 *p* = 0.9 23.3 ± 8.7	*Z* = −1.3 *p* = 0.2 15.9 ± 4.6	*Z* = 0.6 *p* = 0.6 8.4 ± 3.3	*Z* = −0.1 *p* = 0.9 47.6 ± 11.0
	Bad and Mid	35 (41.2%)	42.8 ± 9.5	21.8 ± 5.1	25.0 ± 5.6	21.0 ± 5.0	21.2 ± 5.1	13.7 ± 3.7	16 (45.7%)	23.2 ± 9.1	17.1 ± 4.9	8.0 ± 3.3	48.3 ± 12.2
Income	below minimum wage	67 (78.8%)	*Z* = −0.7 *p* = 0.5 41.6 ± 8.6	*Z* = −1.1 *p* = 0.3 20.9 ± 4.6	*Z* = −0.1 *p* = 0.9 24.3 ± 4.9	*Z* = −0.2 *p* = 0.8 20.6 ± 4.5	*Z* = −0.6 *p* = 0.5 20.3 ± 4.7	*Z* = 0.8 *p* = 0.4 13.5 ± 3.5	*X* = 0.6 *p* = 0.4 39 (58.2%)	*Z* = 0.2 *p* = 0.8 23.4 ± 8.6	*Z* = 0.9 *p* = 0.4 16.6 ± 4.7	*Z* = −0.6 *p* = 0.6 8.1 ± 3.2	*Z* = 0.2 *p* = 0.8 48.1 ± 11.2
	above minimum wage	18 (21.2%)	42.2 ± 9.9	21.8 ± 5.4	23.9 ± 5.9	20.4 ± 4.8	20.8 ± 5.4	12.8 ± 3.3	8 (44.4%)	22.7 ± 9.9	15.5 ± 4.9	8.7 ± 3.5	46.9 ± 12.3
Places	Malatya	82 (96.5%)	*Z* = −0.0 *p* = 1.0 41.7 ± 8.9	*Z* = 0.1 *p* = 0.9 21.0 ± 4.8	*Z* = −0.5 *p* = 0.6 24.1 ± 5.1	*Z* = −0.4 *p* = 0.7 20.5 ± 4.6	*Z* = −0.4 *p* = 0.7 20.4 ± 4.9	*Z* = 0.4 *p* = 0.7 13.3 ± 3.5	*X* = 0.0 *p* = 1.0 45 (54.9%)	*Z* = −0.6 *p* = 0.6 23.1 ± 8.9	*Z* = −0.5 *p* = 0.6 16.3 ± 4.8	*Z* = 0.9 *p* = 0.4 8.3 ± 3.3	*Z* = −0.5 *p* = 0.7 47.8 ± 11.6
	Other	3 (3.5%)	42.3 ± 6.5	21.0 ± 3.6	25.7 ± 4.7	21.7 ± 2.1	21.7 ± 3.8	13.0 ± 2.0	2 (66.7%)	26.0 ± 6.2	18.0 ± 2.0	6.7 ± 2.1	50.7 ± 6.8
Correlations between scales									S.rho = 0.62 *p* < 0.001				Enjoyment: *ρ* = 0.63, *p* < 0.001 P. importance: *ρ* = 0.59, *p* < 0.001 Anxiety: *ρ* = −0.45, *p* < 0.001 *p* < 0.001

**Table 5:** Education level—post-hoc pairwise comparisons, septuagenarian subgroup (Dunn test, Bonferroni correction).

Scale	Group 1	Group 2	Z	p
Sensitivity	Illiterate	Highschool	*Z* = −3.56	*p* = 0.006
Importance	Illiterate	Highschool	*Z* = −4.06	*p* < 0.001
	Primary	Highschool	*Z* = −3.06	*p* = 0.03
Awareness	Illiterate	Highschool	*Z* = −3.17	*p* = 0.02
S. influence	Illiterate	Highschool	*Z* = −4.11	*p* < 0.001
Enjoyment	Illiterate	Primary	*Z* = −3.60	*p* = 0.005
	Illiterate	Highschool	*Z* = −4.84	*p* < 0.001
PSAS	Illiterate	Primary	*Z* = −3.01	*p* = 0.04
	Illiterate	Highschool	*Z* = −4.76	*p* < 0.001

Only statistically significant pairs (*p* < 0.05 after Bonferroni correction) are shown.

**Table 6:** Time with friends or family—post-hoc pairwise comparisons, septuagenarian subgroup (Dunn test, Bonferroni correction).

Scale	Group 1	Group 2	*Z*	*p*
Enjoyment	0–2 days	3–4 days	*Z* = −3.31	*p* = 0.003
P. importance	0–2 days	3–4 days	*Z* = −2.68	*p* = 0.02
PSAS	0–2 days	3–4 days	*Z* = −3.14	*p* = 0.005

Groups: 0–2 days/week, 3–4 days/week, 5 + days/week. Significant pairs only.

### 3.3 Regression analyses

#### 3.3.1 Predictors of OHAS

The multivariate regression model for OHAS total score in the total sample explained 20.1% of the variance (*R*^2^ = 0.201; adjusted *R*^2^ = 0.177; F(8,268) = 8.44, *p* < 0.001). Significant independent predictors were: prior removable denture use (*B* = −13.81; *β* = −0.245; *p* < 0.001), opinion about implant (*B* = 9.73; *β* = 0.132; *p* = 0.026), psychiatric medication use (*B* = −9.34; *β* = −0.133; *p* = 0.021), and education level (*B* = 2.32; *β* = 0.132; *p* = 0.036). In the septuagenarian subgroup, the model explained a higher proportion of variance (*R*^2^ = 0.290; adjusted *R*^2^ = 0.215; *F*(8,76) = 3.88, *p* = 0.001); only prior removable denture use remained a significant predictor (*B* = −17.60; *β* = −0.289; *p* = 0.006). The larger standardized coefficient in the septuagenarian subgroup (*β* = −0.289 vs. − 0.245) indicates that prior denture experience exerts a more pronounced effect on oral health attitudes in older individuals. Full regression results are presented in Table 7.

**Table 7:** Linear regression—predictors of OHAS score: total sample vs. septuagenarian subgroup.

OHAS—oral health attitude scale												
Variable	Total sample (*N* = 277) *R*^2^ = 0.201, Adj. *R*^2^ = 0.177, *F*(8,268) = 8.44, *p* < 0.001, *N* = 277						Septuagenarian subgroup (*N* = 85) *R*^2^ = 0.29, Adj. *R*^2^ = 0.215, *F*(8,76) = 3.88, *p* < 0.001, *N* = 85					
	*B*	*SE*	*β*	*t*	*p*	Sig.	*B*	*SE*	*β*	*t*	*p*	Sig.
Age	0.08	0.27	0.017	0.3	0.766		−0.28	0.6	−0.048	−0.46	0.645	
Gender (Male = 1)	−3.76	3.12	−0.066	−1.21	0.229		8.98	5.99	0.153	1.5	0.138	
Lifestyle (Urban = 1)	6.18	3.43	0.104	1.8	0.073		11.76	6.67	0.197	1.76	0.082	
Prosthetic use (Yes = 1)	**−13.81**	**3.26**	**−0.245**	**−4.23**	***p* < 0.001**	***	**−17.6**	**6.17**	**−0.289**	**−2.85**	***p* < 0.01**	**
Implant opinion (Pos = 1)	**9.73**	**4.34**	**0.132**	**2.24**	***p* < 0.05**	*	13.36	7.71	0.196	1.73	0.087	
Psychiatric med. (Yes = 1)	**−9.34**	**4.03**	**−0.133**	**−2.32**	***p* < 0.05**	*	−10.08	7.09	−0.153	−1.42	0.159	
Education level	**2.32**	**1.1**	**0.132**	**2.11**	***p* < 0.05**	*	−0.69	2.51	−0.036	−0.28	0.783	
Number of implants	−0.34	0.5	−0.037	−0.67	0.506		−0.18	1.04	−0.019	−0.17	0.865	

Method: Enter. Bold = *p* < 0.05. Reference: female, rural, no prosthetic, negative opinion, no psychiatric med. education ordinal 1–6. *p < 0.05, **p < 0.01, ***p < 0.001.

#### 3.3.2 Predictors of PSAS subscales

Separate regression models were constructed for each PSAS subscale; full results are presented in Table 8. For PSAS-Enjoyment in the total sample, the model explained 15.2% of variance (*R*^2^ = 0.152; *F*(8,268) = 6.02, *p* < 0.001); significant predictors were prior denture use (*β* = −0.187, *p* = 0.002), psychiatric medication use (*β* = −0.164, *p* = 0.006), and opinion about implant (*β* = 0.155, *p* = 0.010). For PSAS-Perceived Importance, the model was significant (*R*^2^ = 0.092; *p* < 0.001), with prior denture use (*β* = −0.144, *p* = 0.018) and psychiatric medication use (*β* = −0.151, *p* = 0.014) as significant predictors. The PSAS-Anxiety model was non-significant in the total sample (*R*^2^ = 0.031, *p* = 0.386) and in the septuagenarian subgroup (*R*^2^ = 0.058, *p* = 0.783).

**Table 8:** Linear regression—predictors of PSAS score: total sample vs. septuagenarian subgroup.

Variable	Total sample (*N* = 277)						Septuagenarian subgroup (*N* = 85)					
	*B*	*SE*	*β*	*t*	*p*	Sig.	*B*	*SE*	*β*	*t*	*p*	Sig.
Outcome: enjoyment | Total: *R*^2^ = 0.152, Adj. *R*^2^ = 0.127, *F*(8,268) = 6.02, *p* < 0.001 | Septuagenarian: *R*^2^ = 0.425, Adj. *R*^2^ = 0.365, *F*(8,76) = 7.03, *p* < 0.001												
Age	−0.088	0.076	−0.067	−1.15	0.253		−0.224	0.163	−0.130	−1.37	0.174	
Gender (Male = 1)	0.506	0.894	0.032	0.57	0.572		4.245	1.570	0.242	2.70	*p* < 0.01	
Lifestyle (Urban = 1)	1.817	0.968	0.110	1.88	0.062		2.731	1.723	0.154	1.58	0.117	
Prosthetic use	−2.943	0.920	−0.187	−3.20	*p* < 0.01		−3.696	1.627	−0.204	−2.27	*p* < 0.05	
Implant opinion	3.185	1.220	0.155	2.61	*p* < 0.05		6.118	2.029	0.302	3.02	*p* < 0.01	
Psychiatric med.	−3.211	1.160	−0.164	−2.77	*p* < 0.01		−3.142	1.883	−0.160	−1.67	0.099	
Education level	0.137	0.266	0.030	0.52	0.606		−0.693	0.546	−0.123	−1.27	0.209	
No. of implants	−0.138	0.145	−0.055	−0.95	0.341		−0.166	0.282	−0.059	−0.59	0.559	
Outcome: P. ımportance | Total: *R*^2^ = 0.092, Adj. *R*^2^ = 0.065, *F*(8,268) = 3.41, *p* < 0.001 | Septuagenarian: *R*^2^ = 0.220, Adj. *R*^2^ = 0.138, *F*(8,76) = 2.68, *p* = 0.012												
Age	−0.054	0.047	−0.070	−1.15	0.249		−0.205	0.102	−0.224	−2.02	*p* < 0.05	
Gender (Male = 1)	0.424	0.551	0.045	0.77	0.442		0.655	0.976	0.070	0.67	0.505	
Lifestyle (Urban = 1)	0.416	0.597	0.042	0.70	0.486		0.070	1.072	0.007	0.07	0.948	
Prosthetic use	−1.348	0.567	−0.144	−2.38	*p* < 0.05		−2.231	1.012	−0.230	−2.20	*p* < 0.05	
Implant opinion	1.210	0.751	0.099	1.61	0.108		2.776	1.262	0.257	2.20	*p* < 0.05	
Psychiatric med.	−1.759	0.715	−0.151	−2.46	*p* < 0.05		−0.974	1.171	−0.093	−0.83	0.408	
Education level	0.108	0.164	0.040	0.66	0.512		0.109	0.340	0.036	0.32	0.748	
No. of implants	−0.146	0.089	−0.097	−1.64	0.103		−0.137	0.176	−0.091	−0.78	0.438	
Outcome: anxiety (reverse-coded) | Total: *R*^2^ = 0.031, Adj. *R*^2^ = 0.002, *F*(8,268) = 1.07, *p* = 0.386 (ns) | Septuagenarian: *R*^2^ = 0.058, Adj. *R*^2^ = −0.041, *F*(8,76) = 0.59, *p* = 0.783 (ns)												
Age	−0.014	0.034	−0.025	−0.41	0.684		0.087	0.078	0.137	1.13	0.263	
Gender (Male = 1)	−0.515	0.395	−0.079	−1.30	0.194		−0.059	0.746	−0.009	−0.08	0.937	
Lifestyle (Urban = 1)	−0.515	0.428	−0.075	−1.20	0.230		0.197	0.819	0.030	0.24	0.810	
Prosthetic use	0.423	0.407	0.065	1.04	0.299		0.798	0.773	0.118	1.03	0.305	
Implant opinion	−0.159	0.539	−0.019	−0.30	0.768		−0.733	0.964	−0.098	−0.76	0.449	
Psychiatric med.	0.502	0.513	0.062	0.98	0.328		−0.381	0.894	−0.052	−0.43	0.671	
Education level	−0.085	0.117	−0.045	−0.72	0.471		0.265	0.259	0.127	1.02	0.310	
No. of implants	0.101	0.064	0.096	1.58	0.116		−0.035	0.134	−0.034	−0.26	0.795	

Method: Enter. Bold = *p* < 0.05. Reference categories as in Table 7. *p < 0.05, **p < 0.01, ***p < 0.001.

**Figure 1:**
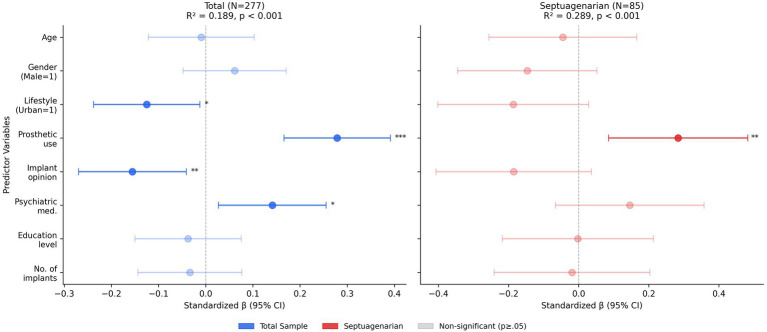
Forest plot - predictors of OHAS score standardized regression coefficients (*ß*) with 95% Cls.

**Figure 2:**
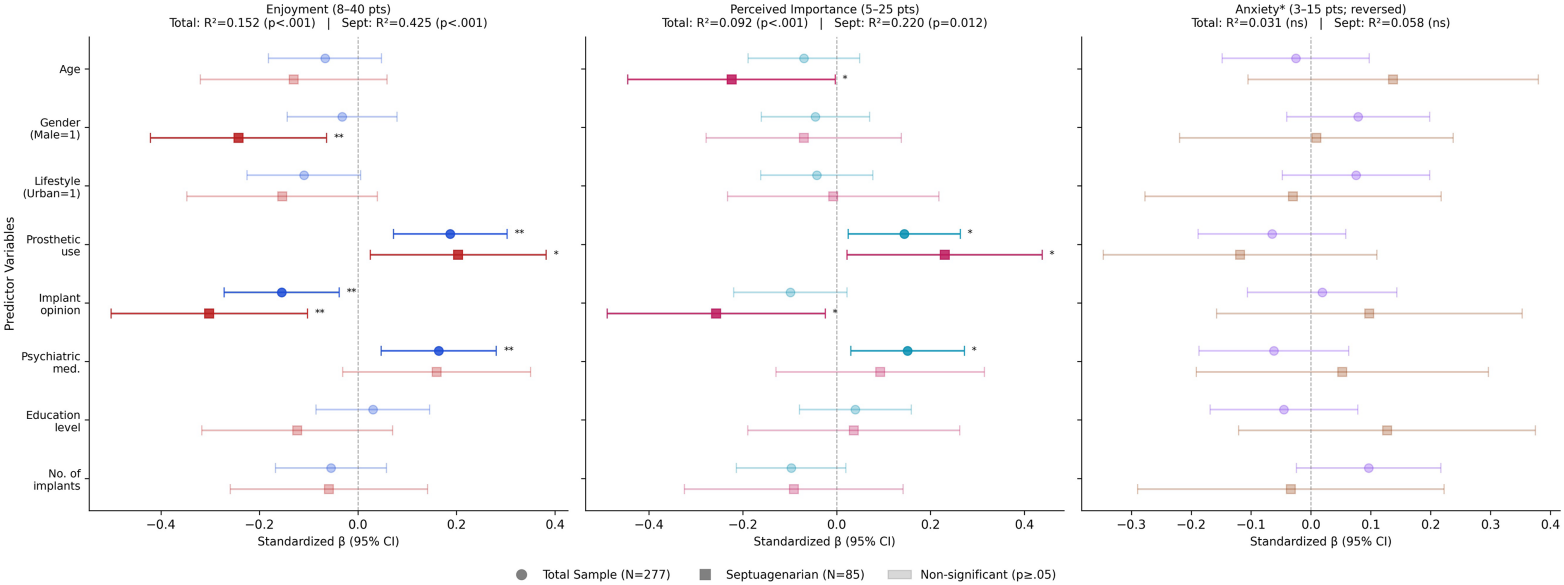
Forest plot - predictors of PSAS subscale scores standardized regression coefficients (*ß*) with 95% CIs.

In the septuagenarian subgroup, the PSAS-Enjoyment model achieved the highest explanatory power (*R*^2^ = 0.425; *F*(8,76) = 7.03, *p* < 0.001); positive opinion about implant (*β* = 0.302, *p* = 0.003) and male gender (*β* = 0.242, *p* = 0.008) were positive predictors, while prior denture use (*β* = −0.204, *p* = 0.026) was a negative predictor. For PSAS-Perceived Importance in this subgroup, age (*β* = −0.224, *p* = 0.047), prior denture use (*β* = −0.230, *p* = 0.030), and opinion about implant (*β* = 0.257, *p* = 0.031) were significant predictors (*R*^2^ = 0.220). The substantially higher *R*^2^ values in the septuagenarian subgroup compared with the total sample (Enjoyment: 0.425 vs. 0.152; Perceived Importance: 0.220 vs. 0.092) suggest that age-specific pathways operate in shaping communication attitudes following implant treatment in the oldest-old. Forest plots depicting *β* coefficients with 95% confidence intervals for all regression models are presented in Figures 1, 2.

## 4 Discussion

### 4.1 Principal findings

This study demonstrated that positive oral health attitudes are significantly associated with public speaking confidence-as measured by PSAS-Enjoyment and PSAS-Perceived Importance-in implant-treated adults aged ≥60 years, with the associations being markedly stronger in the septuagenarian subgroup (≥70 years). These findings are consistent with the literature indicating that successful oral rehabilitation extends beyond masticatory restoration to enhance social interaction capacity and communicative confidence (13, 14). The multidimensional approach of analyzing three PSAS subscales separately revealed that the predictors and model fit for speech anxiety (PSAS-Anxiety) differed substantially from those for enjoyment and importance dimensions, providing insights that a composite score would have obscured.

### 4.2 Findings in the oldest-old septuagenarian subgroup

The most striking finding was that the regression model for PSAS-Enjoyment in the septuagenarian subgroup (*R*^2^ = 0.425) explained approximately three times the variance explained in the total sample (*R*^2^ = 0.152). A similar pattern emerged for PSAS-Perceived Importance (*R*^2^ = 0.220 vs. 0.092). This suggests that sociodemographic and clinical determinants exert increasingly powerful age-specific influences on communication attitudes in the oldest-old. These findings align with prior evidence that the psychosocial impact of oral rehabilitation deepens with advancing age, and that implant success in this group is mediated largely by perceptual processes rather than biological factors alone (15, 16). Several biopsychosocial mechanisms may account for the markedly higher explanatory power observed in the septuagenarian subgroup. First, from a psychological perspective, older adults in the eighth decade and beyond may be more susceptible to the cumulative psychosocial consequences of oral deterioration—including prolonged denture experience and associated self-image disruption—because oral function and appearance have been integral to social identity across a longer life span. In this context, dental implant rehabilitation may represent a qualitatively different psychological event than in younger-old adults, carrying greater symbolic significance as a restoration of communicative agency and social presence. Second, from a social perspective, the networks and roles available to adults aged ≥70 years are often contracted relative to those of adults in their 60’s, rendering each remaining communicative opportunity proportionally more meaningful and more susceptible to attitude-related modulation. This would amplify the association between oral health attitudes and public speaking confidence specifically in the oldest-old. Third, from a biological perspective, age-related changes in phonation, articulation, and facial proprioception may make septuagenarians more cognitively aware of the functional role of their dentition during speech, thereby strengthening the subjective link between oral health attitude and speaking comfort. Fourth, the role of positive implant opinion as the strongest predictor of PSAS-Enjoyment in this subgroup (*β* = 0.302) suggests that psychological readiness and satisfaction with treatment may partially compensate for systemic and social constraints that are more prevalent in advanced age. Collectively, these mechanisms suggest that the relationship between oral rehabilitation and communicative well-being is not linear across the aging spectrum but becomes increasingly consolidated and psychosocially mediated in the oldest-old—a finding with implications for tailoring pre- and post-implant psychological support interventions to patient age.

### 4.3 Non-predictability of speech anxiety

The PSAS-Anxiety subscale regression model was non-significant in both groups (total sample: *R*^2^ = 0.031, *p* = 0.386; septuagenarian subgroup: *R*^2^ = 0.058, *p* = 0.783). These results should be interpreted as inconclusive given severely limited statistical power (1 − *β* = 0.283–0.512); they do not allow conclusions to be drawn regarding the true association between the predictor set and communication anxiety. The non-significance may reflect inadequate power rather than a genuine absence of association. As a descriptive observation, the pattern suggests that the sociodemographic and clinical variables examined-including age, gender, lifestyle, denture history, implant opinion, psychiatric medication use, education level, and number of implants-do not adequately explain variance in communication anxiety. Several interpretations are plausible. First, speech anxiety may be predominantly shaped by chronic biographical variables such as personality traits, dispositional anxiety, and lifetime communication experiences, none of which were captured in this cross-sectional design. Second, the three-item composition of the PSAS-Anxiety subscale may limit its psychometric sensitivity. Third, the restricted score distribution (mean 8.4, SD 3.3; reverse-scored range 3–15) may have attenuated regression power. Future studies should incorporate neuroticism, social anxiety disorder history, and communication self-efficacy as predictors of this dimension. Specifically, an adequately powered investigation of the anxiety dimension in a septuagenarian-only sample would require an estimated minimum of *n* ≈ 200 participants (based on *α* = 0.05, 1 − *β* = 0.80, *f*^2^ = 0.10 for 8 predictors)—a sample size that was not feasible within the present single-center design. Until such a study is conducted, null findings for the PSAS-Anxiety model should not be interpreted as evidence of a genuine absence of association between the tested predictors and communication anxiety in older implant patients.

### 4.4 Gender effects in the septuagenarian subgroup

While gender was not a significant predictor of any PSAS subscale in the total sample, male gender emerged as a significant positive predictor of PSAS-Enjoyment in the septuagenarian subgroup (*β* = 0.242, *p* = 0.008). This finding suggests that, in the oldest-old, men find public speaking after dental implant treatment more enjoyable and fulfilling. This may reflect the persistent influence of gender roles and the historically greater engagement of men in public discourse, which appears to remain salient in advanced age. Alternative explanations include differences in general health attitudes and treatment satisfaction between older men and women. The mechanisms underlying this gender effect warrant further investigation, particularly through qualitative approaches.

### 4.5 Negative correlates: prior denture use and psychiatric medication

Prior removable denture use and psychiatric medication use were consistent negative correlates of both OHAS and PSAS subscales (Enjoyment and Perceived Importance). The stronger negative effect of prior denture use on OHAS in the septuagenarian subgroup (*β* = −0.289 vs. − 0.245) aligns with participant satisfaction research indicating that individuals with prolonged denture experience develop more negative attitudes when implant-related expectations are not fully met (17, 18). The negative associations of psychiatric medication use with PSAS-Enjoyment (*β* = −0.164, *p* = 0.006) and PSAS-Perceived Importance (*β* = −0.151, *p* = 0.014) are consistent with evidence that medication-induced xerostomia and other oral side effects directly constrain social communication comfort (19). Conversely, positive opinion about the implant was the strongest positive predictor of PSAS-Enjoyment in the septuagenarian subgroup (*β* = 0.302, *p* = 0.003), suggesting that psychological readiness and positive attitudes can partially compensate for systemic constraints (20). These findings collectively support a holistic evaluation approach that incorporates medication management, participant expectations, and psychosocial readiness beyond prosthetic outcomes (21).

### 4.6 Sociodemographic and environmental influences

Although education level was not an independent predictor in PSAS regression models, a pronounced attitude gradient by education was observed in group comparisons, particularly in the septuagenarian subgroup, where illiterate individuals scored significantly lower than high school graduates on all relevant subscales. This supports the hypothesis that health literacy facilitates post-treatment adaptation (22). Urban residence and higher social interaction frequency were both associated with higher OHAS and PSAS-Enjoyment scores, suggesting that social environment serves as an independent protective buffer for communication attitudes. This finding has clinical relevance for rural-dwelling and socially isolated older adults, who may require additional psychosocial support following implant treatment.

### 4.7 Comparison with prior work

To our knowledge, no prior study has simultaneously examined oral health attitudes and public speaking confidence in implant-treated older adults using validated multidimensional instruments in both a general older population and a septuagenarian subgroup. Earlier studies have documented associations between oral health status and health-related quality of life (23, 24), and between implant therapy and participant-reported outcomes (25, 26), but the specific pathway through communicative confidence remains unexplored. The finding that OHAS-attitude explains a substantially larger proportion of variance in PSAS-Enjoyment in the ≥70-year group than in the broader ≥60-year group advances the literature by identifying a developmentally specific psychosocial mechanism.

### 4.8 Limitations

Several limitations should be considered. The cross-sectional design precludes causal inference; the direction and temporal sequence of the observed associations cannot be established. The single-center setting (Inonu University Faculty of Dentistry) may limit generalizability to populations with different geographic and socioeconomic profiles. The sample comprised individuals who had successfully accessed implant treatment, potentially excluding older adults with lower income or reduced healthcare access. This constitutes a form of selection bias, as individuals who could not access or afford implant treatment—a group that may include a disproportionate number of rural, low-income, and less educated older adults—are systematically excluded, potentially inflating observed attitude scores relative to the general geriatric population. Additionally, the unequal distribution between the septuagenarian subgroup (*n* = 85; 30.7%) and the 60–69-year group (*n* = 192; 69.3%) reflects the natural age distribution of the clinic population; while this imbalance results in reduced statistical power for subgroup-specific analyses and may constrain the generalizability of septuagenarian-specific findings, *post hoc* power analyses confirmed adequate power (1 − *β* ≥ 0.939) for the significant regression models in this subgroup. The demographic and clinical profile of the septuagenarian subgroup (mean age 74.6 years, 52.9% male, 63.5% prior denture use) is broadly representative of implant-seeking older adults in Turkish tertiary care settings; however, generalizability to community-dwelling or non-implant-treated older adults remains limited. Furthermore, the large number of statistical comparisons conducted in this study inherently increases the risk of Type I error. Given the exploratory, hypothesis-generating nature of this first investigation in septuagenarians, we elected not to apply overly conservative corrections (e.g., Bonferroni) that would substantially increase Type II error risk for novel findings in an understudied population; however, we explicitly acknowledge this as a limitation, and future confirmatory studies will require pre-registered significance thresholds and appropriate alpha adjustment. Self-reported measures are susceptible to social desirability bias. Finally, the three-item composition of the PSAS-Anxiety subscale may have limited statistical power for detecting predictors of this dimension, as reflected in *post hoc* power estimates of 1 − *β* = 0.283–0.512 for the Anxiety models. Future studies should employ longitudinal designs, multicenter samples, and psychometrically richer communication measures. An adequately powered investigation of predictors of communication anxiety in a septuagenarian-only implant sample would require a minimum of approximately 200 participants (*α* = 0.05, 1 − *β* = 0.80, *f*^2^ = 0.10, 8 predictors), which was not achievable within the present single-center design.

## 5 Conclusion

Positive oral health attitudes are significantly associated with public speaking confidence in implant-treated older adults, with the effect being most pronounced in those aged ≥70 years. Prior denture history and psychiatric medication use are consistently negatively associated with both oral health attitudes and communicative enjoyment, whereas positive implant opinion was positively associated with public speaking enjoyment scores in the septuagenarian subgroup. The non-predictability of speech anxiety by sociodemographic and clinical variables underscores the importance of incorporating personality and communication self-efficacy constructs in future research. Clinically, these findings support the adoption of a biopsychosocial protocol in implant care: (1) systematic assessment of prior denture history and psychiatric medication use pre-treatment; (2) evaluation of oral health attitudes using validated instruments such as the OHAS; (3) brief motivational counseling for patients with low attitude scores prior to implant placement; and (4) six-monthly monitoring of social participation and communicative confidence in patients aged ≥70 years.

## Data Availability

The original contributions presented in the study are included in the article/supplementary material, further inquiries can be directed to the corresponding author.
